# Introduction to the special issue of the Consortium of Organizations for Strong Motion Observation Systems (COSMOS) international guidelines for applying noninvasive geophysical techniques to characterize seismic site conditions

**DOI:** 10.1007/s10950-022-10104-w

**Published:** 2022-08-10

**Authors:** Alan Yong, Aysegul Askan, John Cassidy, Sebastiano D’Amico, Stefano Parolai, Marco Pilz, William Stephenson

**Affiliations:** 1 U.S. Geological Survey, Earthquake Science Center, 350 North Akron Road, Moffett Field, Mountain View, CA 94025 USA; 2 Department of Civil Engineering, Middle East Technical University, Ankara, Turkey; 3 Geological Survey of Canada, Pacific Division, Sidney, B.C. and of Earth and Ocean Sciences, University of Victoria, British Columbia, Canada; 4 Department of Geosciences, University of Malta, Msida Campus, Msida, Malta; 5 Istituto Nazionale di Oceanografia e di Geofisica Sperimentale, OGS, Sgonico, TS Italy; 6 Deutsches GeoForschungsZentrum, Potsdam, Germany; 7 U.S. Geological Survey, Golden, Colorado USA

**Keywords:** COSMOS guidelines, Best-practices, Seismic site characterization, Earthquake site effects

## Introduction

Knowledge about local seismic site conditions provides critical information to account for site effects that are commonly observed in strong motion recordings. Certainly, other wave propagation effects can influence these observations, which are attributable to variations in material properties of the paths traveled by the waves, as well as the characteristics of the seismic source. However, local geologic conditions, particularly, when under shear-wave excitation, are known to have a strong influence on the behavior of ground shaking in the frequency range that is expected to directly affect the built environment. Thus, shear waves traveling in the shallow subsurface—defined here as tens to hundreds of meters beneath the ground surface—are the main foci for application and research in the earthquake engineering community.

To assess the potential for important site effects, a number of approaches collectively known as site response analyses (SRA) are constantly developed. They are also continuously tested and refined with the aim to reduce the uncertainties associated with each technique. Although SRA can be carried out empirically, a set of popular procedures within the suite of SRA methods relies on numerical techniques (one dimensional [1D] transfer functions) and is further differentiated by earthquake engineers as ground response analysis (GRA). Fundamentally, GRAs require input from measurements through in situ seismic recordings that are generally known as the field data acquisition component of site characterization. Following such acquisitions are the associated data processing and analysis phases that produce the shear-wave velocity (*V*_*S*_) profile as the main output, as well as its derivative, the time-averaged *V*_*S*_ of the upper 30 m from the surface (*V*_*S*30_), which is the main site index term in ground motion modeling (Boore et al. 1993; Borcherdt 1994).

To advance knowledge about site effects phenomena, special SRA-focused sessions have become common occurrences at internationally held earthquake conferences and scientific journals have frequently devoted special issues (or sections) to document the state of the knowledge (Field et al. 2000; Panzera et al. 2017; Kaklamanos et al. 2021). Recently, Kaklamanos et al. (2021) introduced a collection of papers compiled as a special section entitled *Advancements in Site Response Estimation*, which originated from a similarly named special session planned for the 2020 Annual Meeting of the Seismological Society of America (which was canceled due to the COVID-19 pandemic). Through open submissions, the guest editors organized articles into five interrelated sections about various aspects of site response (Kaklamanos et al. 2021), including five papers addressing uncertainties as contributed through the SRA framework, as well as one general section on site characterization. Of the six papers included in this section, only two were primarily focused on *V*_*S*_ measurements and both focused on the use of surface wave methods to generate in situ *V*_*S*_ models (Hobiger et al. 2021; Stephenson et al. 2021). The study locations of each paper were unrelated, but both papers shared the general approach of comparing surface-wave-based analytical estimates of the site dominant frequencies (*f*_*d*_) to that of earthquake horizontal-to-vertical spectral ratios (eHVSR). These independent studies found strong agreement between their modeled and observed *f*_*d*_. In a more recent effort, S. Matsushima and others (http://www.esg6.jp/blind.html; last accessed 4 April 2022) conducted blind tests that were mainly focused on SRA through participation by international analysts as part of the 2021 6th International Symposium of the Effects of Surface Geology on Seismic Motion.

During the past two decades, advancements in the field of site characterization have also benefited from activities that were similarly conducted for SRA. This period coincided with a time when applying cost-effective noninvasive surface-wave approaches gained tremendous popularity worldwide. Particularly important were related crossover efforts that attempted to assess uncertainties propagated from methodologies that apply surface-based site characterization to GRAs. To this end, a number of blind trials on-site characterization methods were conducted and most of these activities were directly followed with developments of guidelines for best practices by organizers of the trials (Cornou et al. 2007; Boore and Asten 2008; Garofalo et al. 2016; Foti et al. 2018; Asten et al. 2022, this issue). Unassociated guidelines, technical reports, and textbooks about the application of surface wave methods were also independently published by authors and many were participants of the aforementioned trials (SESAME 2004; Yong et al. 2013; Martin et al. 2014; Dal Moro 2014; Foti et al. 2015; Martin et al. 2017). Despite these accomplishments, the findings illuminated solutions, which also inherently beget more questions, and thus the continuation of these activities is expected for the foreseeable future (Askan et al. 2022).

## COSMOS guidelines

The COSMOS International Guidelines for Applying Noninvasive Geophysical Techniques to Characterize Seismic Site Conditions Project is an effort that was borne through the consensus of the international site characterization and site response research communities. This mixture of for- and non-profit entities is (to date) the most comprehensive collection of practitioners of near-surface geophysics and/or seismic hazards analyses, globally recognized key developers of the state of practice in geophysical site characterization methods, and research scientists from academic and governmental institutions (Table 1). The COSMOS Site Characterization Project (title contracted for brevity here and henceforth) was conceived during its inaugural 24 April 2015 workshop (post-2015 Annual Meeting of the Seismological Society of America, Pasadena, CA, USA), when more than 80 members of the seismological and engineering research communities participated. At the close of the workshop, attendees unanimously decided to form a focus group with the goal of facilitating the development of guidelines for and addressing issues relating to aleatoric and epistemic uncertainties in noninvasive surface-based site characterization approaches. To initiate this effort, the COSMOS Site Characterization Project Committee was assembled (Table 1) to convene workshops and special sessions at various international conferences. A main outcome of the activities of this COSMOS committee is the compilation of papers in this special issue. This paper serves as the introduction to this special issue, as well as a summary report of the COSMOS activities from 2015 to 2022 leading to these publications.

**Table 1:** Consortium of Organizations for Strong Motion Observation Systems Facilitation Committee Members

**Member**	**Role**	**Country**
Alan Yong	Chair-person (2015–2021)	USA
Jamison Steidl	Vice chair-person (2015–2021)	USA
Robert Nigbor	Vice chair-person (2015–2021)	USA
William Stephenson	Chair-alternate (2015–2021)	USA
Aysegul Askan	Co-chair-person (2021–present)	Turkey
Sebastiano D’Amico	Co-chair-person (2021–present)	Malta
Marco Pilz	Co-chair-person (2021–present)	Germany
Michael Asten	Representative	Australia
Sheri Molnar	Representative	Canada
John Cassidy	Representative	Canada
Heather Crow	Representative	Canada
Martin Lawrence	Representative	Canada
Jianghai Xia	Representative	China
Cécile Cornou	Representative	France
Pierre-Yves Bard	Representative	France
Fabrice Hollender	Representative	France
Stefano Parolai	Representative	Italy
Sebastiano Foti	Representative	Italy
Laura Socco	Representative	Italy
Dino Bindi	Representative	Germany
Hiroshi Kawase	Representative	Japan
Shinichi Matsushima	Representative	Japan
Hiroaki Yamanaka	Representative	Japan
Liam Wotherspoon	Representative	New Zealand
Ruben Boroschek	Representative	South America
Donat Fäh	Representative	Switzerland
Jeff Bachuber	Representative	USA
Matthew Muto	Representative	USA

### Summary of Articles in this Volume

The papers in this COSMOS special issue were originally intended as a set of instructional materials for participants in a COSMOS site characterization and site response blind trial planned over 2019–2022. This COSMOS trial should not be confused with the earlier 2018 COSMOS site characterization (microtremor array only) trial by Asten et al. (2022; in this issue), which was intended as preparation for the multi-year blind trial. In the crossover trial, participants would analyze noninvasive active- and passive-source surface- and body-wave data recorded at undisclosed locations. The locations were chosen to represent a variety of seismic monitoring stations that recorded earthquakes and a variety of seismic site conditions ranging from hard rock to soft soils. Moreover, locations that represent the intermediate site conditions between rock and soil sites were known to have the potential to induce various types of complex ground shaking behaviors (shallow impedance layers below the soil; lateral velocity variations; anisotropic effects; etc.). A phased release of site information was planned and participants were to consist of international analysts, who are separated into three tiers that define the individual’s (or team’s) a priori knowledge: level 1, students (advanced undergraduate to graduate education); level 2, strictly commercial industry practitioners (little to no research experiences); and level 3, advanced professional experts and academic researchers. Goals for field data acquisition and the content of instructional materials were partially accomplished in 2019–2020. The blind tests would have been conducted during the rest of 2020. Then, evaluations of the test results would have been performed and presented in 2021, followed by the publication of the COSMOS site characterization guidelines based on empirical evidence to support estimates of epistemic and aleatoric uncertainties, as well as inter- and intra-analyst biases, as computed from results of the blind trial.

However, in May 2020, the blind trial was interrupted by the COVID-19 pandemic, which prompted the COSMOS Committee to reluctantly cancel the test components and revise the plans by focusing on the ongoing efforts to develop instruction sets for a journal special issue focused solely on the state of knowledge and practice for noninvasive site characterization methods. To this end, eleven papers were curated by the COSMOS guest editors. These articles are assigned to three main themes: the first topic articulates the best practice for applications of various site characterization methods (Louie et al. 2022; Pancha and Apperley 2022; Hayashi et al. 2022; Hunter et al. 2022; Molnar et al. 2022; Stephenson et al. 2022); the second is agnostic to the aforementioned techniques and focuses on processing and analyzing data (Toro 2022; Vantassel and Cox 2022), including one paper on the role of analysts (Asten et al. 2022); and the third involves reviews of select topics that are fundamental for consideration in all techniques (Gosselin et al. 2022; Parolai et al. 2022). All papers are aligned on issues relating to uncertainty, which are paramount to the practice of site characterization as performed at the time of publication. These thematic papers are indexed herein by the key words: “COSMOS Guidelines,” or some combination of the terms “COSMOS” and “guidelines.”

#### Site characterization methods

Surface-based site characterization methods differ mostly in their data acquisition approach but share similar procedures for data processing and analyses (Socco et al. 2010; Foti et al. 2014; Yong et al. 2019). Thus, the section addressing the application of techniques includes six select papers that can be generally categorized into two types: standalone single-method approaches (Louie et al. 2022; Pancha and Apperley, 2022; Hayashi et al. 2022; Hunter et al. 2022; Molnar et al. 2022) and the so-called multi-method approaches (Stephenson et al. 2022). Within the standalone approach, each method can also be differentiated by whether it relies on either active- or passive-source energy. The former is based on the controlled generation of seismic waves and the latter is through a mixture of anthropogenic and naturally occurring ambient noise or microtremors. Furthermore, whether a method is either a single- or multi-station array approach is an important distinction that is related to field acquisition procedures. Thus, this section is first organized on the basis of whether the site characterization technique is a standalone single-method or a multi-method approach; next, the approach is categorized by whether it is reliant on active- or passive-source energy; then, a final distinction is made based on whether a single- or multi-station technique is used.

For shear wave (horizontally polarizing only) reflection and refraction methods, Hunter et al. (2022, this issue) review the current state of practice, as well as provide discussions about the limitations and associated uncertainties inherent in this traditional set of surface-based body-wave techniques. This paper begins with data collection, then navigates through a course on basic and advanced steps to process and analyze the data. Methods are described that use various energetic sources capable of generating a wide range of frequencies (including manual hammer strikes and mechanical sweeps of frequency ranges from mini and larger vibration sources) that rely on traditional stationary array recordings and that use mobilized landstreamers. Case studies reflecting a range of site conditions at eight locations in North America are used to support the remarks and recommendations by Hunter et al. (2022).

There are other commonly applied active-source techniques not included in this special issue, in part, because they are already well-described in the literature. Notably, both spectral analysis of surface waves (SASW) (Stokoe and Nazarian 1985) and multichannel analysis of surface waves (MASW) (Park et al. 1998; Foti 2000) are universally used for site characterization at multiple scales. Although these surface-wave methods differ greatly from body-wave methods as addressed by Hunter et al. (2022), applications of both the SASW and MASW techniques have been satisfactorily addressed in the literature by the original developers, who provided detailed and consistent guidance about procedures through a vast number of publications (Stokoe and Nazarian 1985, 1985; Stokoe et al. 1994, 2004, 2017, 2019; Park et al. 1998, 1999, 2000, 2005; Foti 2000), as well as from the direct outcome of blind trials (Garofalo et al. 2016; Foti et al. 2018), or as a complementary technique within a multi-method framework (Park et al. 2005; Yong et al. 2013, 2019; Stephenson et al. 2022, in this issue). Moreover, the commercial market is replete with offerings of sophisticated software packages capable of analyses using various types of active- (and passive-) source techniques. Thus, the addition of addressing the SASW and MASW methods is not included in this issue.

Through the framework of a crossover paper about site response and site characterization, Molnar et al. (2022, this issue) address the state of knowledge and practice on the single-station microtremor horizontal-to-vertical spectral ratio (mHVSR) technique. Using interrogatives like what and how as their foundation, this paper begins with a review of the historical development of the mHVSR technique and consolidates the state of knowledge about the physical basis of a mHVSR (the what), then summarizes recommendations for mHVSR acquisition and analysis (the how), as well as addresses uncertainty therein. Notably, some of the key contributors to this latest mHVSR effort—as well as those in the predecessor (Molnar et al. 2018)—were also principals of the seminal Site Effects Assessment Using Ambient Excitations (SESAME 2004) project. As this type of analysis is now arguably one of the most common site characterization methods worldwide, practitioners have mostly been relying on the SESAME (2004) guidelines. However, the SESAME guidelines are quickly approaching their twentieth year since publication and had been without updates despite advancements in the related sciences and technologies. Thus, recent efforts to improve the SESAME guidelines have gained traction (Wang et al. 2022). To this end, Molnar et al. (2022) take a snapshot of the state of knowledge and add perspectives from their new contributors to document emerging advancements that followed the release of the original Molnar et al. (2018) review.

Hayashi et al. (2022; this issue) review a suite of passive-source two-dimensional (2-D) array analysis techniques that have been referred to as microtremor array methods (MAMs), though other less common combinations of these terms exist in the literature (array microtremor methods, microtremor survey methods, etc.) (Okada 2003; Yong et al. 2013). This paper focuses on the spatial autocorrelation or spatially averaged coherency (SPAC) processing method that is used to analyze recordings from receiver arrays commonly configured in symmetrical forms. For example, these methods use receivers at equidistant spacings, in circular and triangular layouts, with a common center receiver. For larger apertures, progressively larger circles and triangles are nested and in the case of the triangles, each is progressively inverted (Aki 1957). Another MAM-related approach known as the frequency-wavenumber (*f*-*k*) method exists, but it has been deemed more complicated and less stable by the authors, as well as others (Zhao and Li 2010; Asten and Hayashi 2018; Asten et al. 2022, this issue), thus applications of the *f*-*k* method are not addressed herein. Also excluded are analyses of Love wave data due to the ubiquity of Rayleigh wave recordings. Variants of the SPAC approach—such as the extended spatial autocorrelation (ESAC or ESPAC), wavenumber (*k*), and separation (*r*) of station pairs spatial autocorrelation (*kr*SPAC), and multimode or direct-fitting spatial autocorrelation (MMSPAC)—are addressed. Summarily, Hayashi et al. (2022) provide a synopsis of the fundamental principles for determining the Rayleigh-wave phase velocity from ambient noise sources; general recommendations are provided for field data acquisition and limitations and uncertainties of the MAMs are discussed. An earlier review effort by Asten and Hayashi (2018) was the genesis for the COSMOS 2018 blind trial (Asten et al. 2022, in this issue), whose findings are included in Hayashi et al. (2022).

Louie et al. (2022, in this issue) and Pancha and Apperley (2022, in this issue) present a pair of papers focusing on the refraction microtremor (ReMi) technique. The Louie et al. (2022) paper addresses common misapplications (“abuses”) of this popular North American site characterization method that had been in practice since it was introduced by Louie (2001). In two parts, this paper first provides best practices in the form of guidelines and cautions against mistakes by avoidance of pitfalls inherent in all three site characterization phases (acquisition, processing, and analysis); the second half is devoted to discussions on extending the resultant 1D ReMi profiles into 2D cross sections, as well as on deep ReMi surveys to estimate the so-called depth parameters known as *Z*_*n*_ (e.g., when *n* = 1.0, then *Z*_1.0_ is the designation for depth to the 1 km/s iso-surface). As an ancillary to Louie et al. (2022), Pancha and Apperley (2022) draw on three case studies to discuss the utilities of the ReMi technique, including applications of the ReMi technique to map lateral subsurface variations and model seismic velocities between downhole measurements.

Through several case studies, Stephenson et al. (2022, this issue) provide a comprehensive review and assessment of the use of flexible combinations of multiple complementary methods that are optimized for seismic site characterization. Examples of variations of the multi-method approach in increasingly challenging settings are shown. The various strategies based on decision processes for integrating these methods for various challenging geological site conditions, as well as site access issues, are discussed and appropriate background and motivations for each approach are demonstrated. Incorporated in their multi-method approach are all of the standalone active- or passive-source body- or surface-wave site characterization methods compiled in this special issue (Hayashi et al. 2022; Hunter et al. 2022; Louie et al. 2022; Molnar et al. 2022; Pancha and Apperley 2022), in addition to techniques such as MASW and SASW. The authors describe the importance of analyzing both Rayleigh and Love wave dispersion as part of a multi-method approach. This paper provides a general rule-of-thumb for practitioners seeking advice on how to increase the accuracy and reliability of seismic site characterization, with an eye on maintaining cost-effectiveness.

#### Uncertainty in processing and analyzing data

Toro (2022, this issue) introduces the data processing and analysis section by addressing uncertainty in estimating *V*_*S*_ with a simulation model that was primarily developed and refined by Toro (1995, 1997, 2005). Toro (2022) begins with considerations about the two main types of uncertainty, the uncertainty related to intrinsic randomness (aleatoric) and related to lack of knowledge (epistemic), which are considered in probabilistic seismic hazard analysis. Next, issues about the distinction between aleatoric and epistemic uncertainty are discussed. Then, the Toro randomization model (Toro 1995, 1997) is described as applied for generic and site-specific studies. Discussions about recently developed randomization models addressing uncertainty in estimating *V*_*S*_ follow. A brief survey of uncertainty relating to the use of surface-wave methods as addressed by recent publications is summarized to tie these stochastic approaches to the state of practice. As concluding remarks, Toro (2022) provides a suite of recommendations to address uncertainty when implementing probabilistic models in generic and site-specific applications.

Vantassel﻿ and Cox (2022, this issue) also address aleatoric and epistemic uncertainty in estimating *V*_*S*_ by presenting an open-source Python package called SWprocess and associated Jupyter workflows (Vantassel 2021). SWprocess is designed for processing and analyzing surface-wave dispersion data. This paper describes the principles encapsulated in the software, which can also be applied without the use of the package to robustly measure uncertainties. Data processing and statistical analyses were incrementally tested and are incorporated and discussed by Vantassel and Cox (2022). Factors to be considered for the best possible estimates of both active- and passive-source surface-wave dispersion data are illustrated in detail. Throughout the paper, a variety of unique data sets are used to provide the practitioner with real-world examples of the variations of complex issues. For active- and passive-source data sets, as well as the combination of both, step-by-step procedures to quantify dispersion uncertainty, are provided. To encourage the adoption of their recommendations, Vantassel and Cox (2022) direct the practitioner to the readily available SWprocess software. 

Asten et al. (2022, this issue) close this section by presenting their findings from the phased 2018 COSMOS blind trials. In the span of about a year, thirty-four analysts—consisting of individuals and teams and comprising of a wide range of experiences (graduate-level students to professional and academic experts)—participated in the estimation of *V*_*S*_ primarily based on MAM data. Geophysical and geological data recorded and collected at four undisclosed sites with geomorphology ranging from deep alluvial basins to an alpine valley were released in four phases (Asten et al. 2021). Each step was presented with gradually increasing site information to the analysts, particularly array recordings: (1) two-station arrays, (2) sparse triangular arrays, (3) complex nested triangular or circular arrays, and (4) all compilations of geologic control information of the sites, including downhole data. Analysts were encouraged to apply their choice of processing and analysis techniques (beam-forming, cross-correlation, seismic interferometry, or SPAC) using customized and/or commercial software, which allows comparisons of the effectiveness of differing wave field distributions and techniques. To quantitatively compare *V*_*S*_ profiles from multiple analysts, Asten et al. (2022) develop the *M* quality index, which is defined using estimates of the time-averaged *V*_*S*_ of the upper 10 (*V*_*S*10_), 30 (*V*_*S*30_), 100 (*V*_*S*100_), and 300 (*V*_*S*300_) meters from the surface. Asten et al. (2022) conclude that—subject to a sufficient azimuthal distribution of the passively acquired seismic noise sources—the use of sparse arrays is adequate for accurate estimates of *V*_*S*10_, *V*_*S*30_, *V*_*S*100_, or *V*_*S*300_; no techniques/software packages are observed to outperform others for any portion of the trial; and that analyst skill and experience is a stronger factor than that of technique and software choices.

#### Special topics

Gosselin et al. (2022, this issue) review common inversion approaches applied in nearly all active- and passive-source surface-wave dispersion-based site characterization methods. Tomography and full-waveform inversion techniques are also discussed but are restricted to the context of surface waves. Thus, this paper does not address active-source tomographic body-wave methods (Sheehan et al. 2005) as they are formulated based on different governing wave-propagation equations than surface-wave dispersion approaches. A theoretical overview of inversion methods is given first, then followed by discussions about the forward problem, errors and misfits, parameterization, and practical considerations. Next, a wide range of algorithms that are categorized as either linearized (local searches) or nonlinear (optimization) methods are covered, as well as discussions about the probabilistic method based on Bayesian inference with examples to demonstrate the use of the Bayesian information criterion. Propagating uncertainty in analyses of *V*_*S*_ to that of site characterization is an important advantage of the Bayesian approach and aligns directly with the theme of this COSMOS issue.

Parolai et al. (2022, this issue) provide a detailed review of the seismic quality (attenuation) factor, known as *Q* and, in particular, the methodologies by which the shear wave quality factor (*Q*_*S*_) are estimated in common practice. Beginning with considerations about the importance of this parameter in seismology, the paper then proceeds by reflecting on various theoretical definitions of *Q*, as well as the primary wave quality factor (*Q*_*P*_) and *Q*_*S*_. Next, the authors provide a review of the literature on *Q*_*S*_ estimation methods that use data from surface and borehole sensor recordings. Distinctions between active- and passive-source approaches, along with their advantages and disadvantages, and the state of the practice are discussed. A summary of the phenomena associated with the high-frequency shear-wave attenuation factor (kappa) and its relation to *Q*, as well as other lesser-known attenuation parameters are then presented. Parolai et al. (2022) is the final paper of the special topics section of this special issue on site characterization.

## Discussions and concluding remarks

This preface introduces the special issue, consisting of the eleven invited papers curated by select members of the Facilitation Committee (Table 1) for the COSMOS International Guidelines for Applying Noninvasive Geophysical Techniques to Characterize Seismic Site Conditions Project. Since 2015, the COSMOS Site Characterization Project has been working to develop practical guidelines and recommendations through the consensus of the international site characterization and site response research community. The main method of the project has been workshops and special sessions at major international conferences. Despite the impacts of the COVID-19 pandemic, the papers on this special issue represent a major milestone for the COSMOS Site Characterization Project. They include state-of-the-art reviews of geophysical site characterization methods by the developers as well as articles by experts on special topics relating to uncertainty as propagated from site characterization to site response analysis. This work will naturally bring about more questions and the need to continue these activities for the foreseeable future (Askan et al. 2022). Going forward, the COSMOS Facilitation Committee (Table 1) will lead the project to continue to act as an interdisciplinary medium to bring together the engineering and earth science communities associated with site characterization and site response analysis.

## Data Availability

Not applicable.
